# Poly[tetra­kis­(seleno­cyanato-κ*N*)bis­(methanol-κ*O*)tris­(μ-pyrimidine-κ^2^ *N*:*N*′)dicobalt(II)]

**DOI:** 10.1107/S160053681002060X

**Published:** 2010-06-05

**Authors:** Mario Wriedt, Inke Jess, Christian Näther

**Affiliations:** a Institut für Anorganische Chemie, Christian-Albrechts-Universität Kiel, Max-Eyth-Strasse 2, 24098 Kiel, Germany

## Abstract

In the title compound, [Co_2_(NCSe)_4_(C_4_H_4_N_2_)_3_(CH_3_OH)_2_]_*n*_, the Co^II^ ion is coordinated by three N-bonded pyrimidine ligands, two N-bonded seleno­cyanate anions and one O-bonded methanol mol­ecule in an octa­hedral coordination mode. The asymmetric unit consists of one Co^II^ ion, one pyrimidine ligand, two seleno­cyanate anions and one methanol mol­ecule in general positions as well as one pyrimidine ligand located around a twofold rotation axis. In the crystal structure, the pyrimidine ligands bridge [Co(CNSe)_2_(CH_3_OH)] units into zigzag-like chains, which are further connected by pyrimidine ligands into layers parallel to (010).

## Related literature

For general background, see: Wriedt & Näther (2009*a* ▶,*b* ▶); Wriedt, Sellmer & Näther (2009*a* ▶,*b* ▶). For the isotypic structure of a nickel thio­cyanato complex, see: Wriedt *et al.* (2009 ▶).
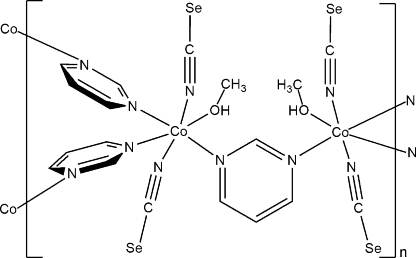


## Experimental

### 

#### Crystal data

[Co_2_(CNSe)_4_(C_4_H_4_N_2_)_3_(CH_4_O)_2_]
*M* *_r_* = 421.07
Orthorhombic, 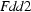

*a* = 20.4069 (8) Å
*b* = 33.0633 (15) Å
*c* = 8.3750 (3) Å
*V* = 5650.8 (4) Å^3^
*Z* = 16
Mo *K*α radiation
μ = 6.36 mm^−1^
*T* = 293 K
0.16 × 0.11 × 0.02 mm

#### Data collection

Stoe IPDS-2 diffractometer
Absorption correction: numerical (*X-SHAPE* and *X-RED32*; Stoe & Cie, 2002 ▶) *T*_min_ = 0.431, *T*_max_ = 0.885
18545 measured reflections
3391 independent reflections
3067 reflections with *I* > 2σ(*I*)
*R*_int_ = 0.044

#### Refinement

*R*[*F* ^2^ > 2σ(*F* ^2^)] = 0.038
*wR*(*F* ^2^) = 0.069
*S* = 1.16
3391 reflections
164 parameters
1 restraint
H-atom parameters constrained
Δρ_max_ = 0.55 e Å^−3^
Δρ_min_ = −0.35 e Å^−3^
Absolute structure: Flack (1983 ▶), 1579 Friedel pairs
Flack parameter: 0.057 (13)

### 

Data collection: *X-AREA* (Stoe & Cie, 2002 ▶); cell refinement: *X-AREA*; data reduction: *X-AREA*; program(s) used to solve structure: *SHELXS97* (Sheldrick, 2008 ▶); program(s) used to refine structure: *SHELXL97* (Sheldrick, 2008 ▶); molecular graphics: *SHELXTL* (Sheldrick, 2008 ▶); software used to prepare material for publication: *SHELXTL*.

## Supplementary Material

Crystal structure: contains datablocks I, global. DOI: 10.1107/S160053681002060X/hy2313sup1.cif

Structure factors: contains datablocks I. DOI: 10.1107/S160053681002060X/hy2313Isup2.hkl

Additional supplementary materials:  crystallographic information; 3D view; checkCIF report

## Figures and Tables

**Table 1:** Selected bond lengths (Å)

Co1—N1	2.191 (3)
Co1—N2^i^	2.188 (3)
Co1—N11	2.184 (3)
Co1—N21	2.064 (4)
Co1—N31	2.059 (4)
Co1—O41	2.142 (3)

Symmetry code: (i) 
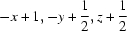
.

**Fig. 1.**
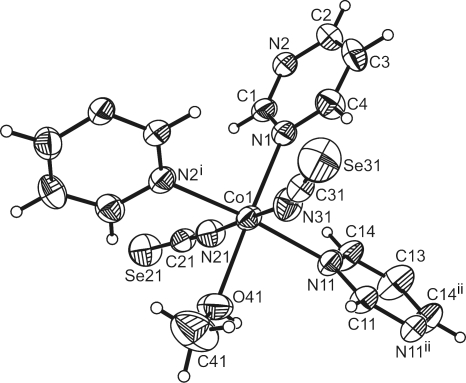
Structure of the title compound with displacement ellipsoids drawn at the 50% probability level. [Symmetry codes: (i) -x+1, -y+1/2, z+1/2; (ii) -x+3/2, -y+1/2, z.]

**Fig. 2.**
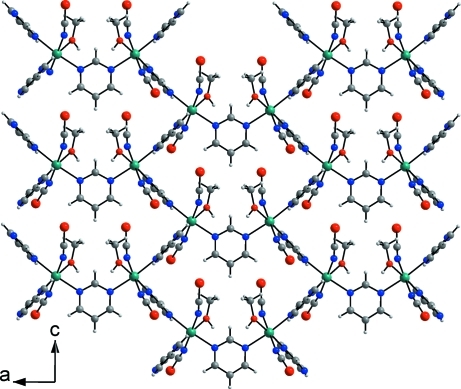
A single layer in the title compound viewed along the b axis.

**Fig. 3.**
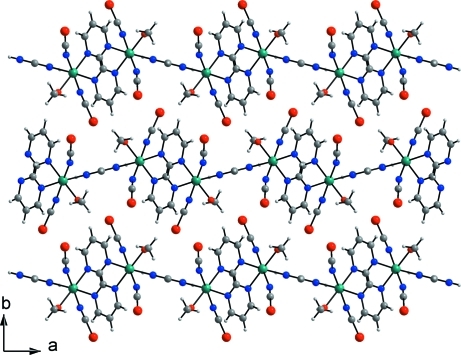
Packing of the layers in the title compound viewed along the c axis.

## supplementary crystallographic information

### Comment

Recently, we have shown that thermal decomposition reactions are an elegante route for the discovering and preparation of new ligand-deficient coordination polymers with defined magnetic properties (Wriedt & Näther, 2009a,b; Wriedt, Sellmer & Näther, 2009a,b). In our ongoing investigation in this field, we have reacted cobalt(II) nitrate, potassium selenocyanate and pyrimidine in methanol. The crystals obtained were identified by single crystal X-ray determination.

The title compound (Fig. 1) represents a two-dimensional layered coordination polymer, which is isotypic to its corresponding nickel(II) thiocyanate analogue reproted recently (Wriedt *et al.*, 2009). The crystal structure consists of µ-1,3-(*N*,*N*) pyrimidine bridged zigzag-like cobalt(II) selenocynate chains, which are further linked by µ-1,3-(*N*,*N*) pyrimidine ligands into layers (Fig. 2). Within each layer the Co^II^ ions are bridged by three pyrimidine ligands and are further terminally coordinated by two *N*-bonded selenocyanate anions and one *O*-bonded methanol molecule in an octahedral coordination mode (Fig. 1). The layers are stacked in the direction of the crystallographic *b*-axis (Fig. 3). The CoN_5_O octahedron is markedly distorted with three long Co—N_pyrimidine_ distances in the range of 2.184 (3) to 2.191 (3) Å, one long Co—O_MeOH _distance of 2.142 (3) Å and two short Co—NCSe distances of 2.059 (4) and 2.064 (4) Å (Table 1). The angles arround the metal centers range between 86.37 (13) to 96.00 (13) and 173.71 (13) to 177.16 (14)°. The shortest intra- and interlayer Co···Co distances amount to 6.0723 (6) and 8.5630 (9) Å, respectively.

### Experimental

Co(NO_3_)_2_.6H_2_O (72.8 mg, 0.25 mmol), KNCSe (72.0 mg, 0.5 mmol) and pyrimidine (20.0 mg, 0.25 mmol) obtained from Alfa Aesar were transfered in a closed snap-vial with methanol (3 ml). After several days at room temperature without stirring, light pink block-shaped single crystals of the title compound were obtained in a mixture with unknown phases.

### Refinement

The O-bound H atom was located in a difference Fourier map and its bond length set to a ideal value and finally refined using a riding model. All other H atoms were positioned with idealized geometry and refined using a riding model, with C—H = 0.93 (CH) and 0.96 (CH_3_) Å and with *U*_iso_(H) = 1.2(1.5 for methyl)*U*_eq_(C). The absolute structure was determined on the basis of 1579 Friedel pairs.

### Crystal data

**Table:**

[Co_2_(CNSe)_4_(C_4_H_4_N_2_)_3_(CH_4_O)_2_]	*F*(000) = 3232
*M**_r_* = 421.07	*D*_x_ = 1.980 Mg m^−^^3^
Orthorhombic, *F**d**d*2	Mo *K*α radiation, λ = 0.71073 Å
Hall symbol: F 2 -2d	Cell parameters from 18545 reflections
*a* = 20.4069 (8) Å	θ = 2.4–28.0°
*b* = 33.0633 (15) Å	µ = 6.36 mm^−^^1^
*c* = 8.3750 (3) Å	*T* = 293 K
*V* = 5650.8 (4) Å^3^	Block, light pink
*Z* = 16	0.16 × 0.11 × 0.02 mm

### Data collection

**Table:**

Stoe IPDS-2 diffractometer	3391 independent reflections
Radiation source: fine-focus sealed tube	3067 reflections with *I* > 2σ(*I*)
graphite	*R*_int_ = 0.044
ω scans	θ_max_ = 28.0°, θ_min_ = 2.4°
Absorption correction: numerical (*X-SHAPE* and *X-RED32*; Stoe & Cie, 2002)	*h* = −26→26
*T*_min_ = 0.431, *T*_max_ = 0.885	*k* = −43→43
18545 measured reflections	*l* = −11→10

### Refinement

**Table:**

Refinement on *F*^2^	Secondary atom site location: difference Fourier map
Least-squares matrix: full	Hydrogen site location: inferred from neighbouring sites
*R*[*F*^2^ > 2σ(*F*^2^)] = 0.038	H-atom parameters constrained
*wR*(*F*^2^) = 0.069	*w* = 1/[σ^2^(*F*_o_^2^) + (0.0286*P*)^2^ + 8.8704*P*] where *P* = (*F*_o_^2^ + 2*F*_c_^2^)/3
*S* = 1.16	(Δ/σ)_max_ < 0.001
3391 reflections	Δρ_max_ = 0.55 e Å^−^^3^
164 parameters	Δρ_min_ = −0.35 e Å^−^^3^
1 restraint	Absolute structure: Flack (1983), 1579 Friedel pairs
Primary atom site location: structure-invariant direct methods	Flack parameter: 0.057 (13)

### Fractional atomic coordinates and isotropic or equivalent isotropic displacement parameters (Å^2^)

**Table:**

	*x*	*y*	*z*	*U*_iso_*/*U*_eq_	
Co1	0.60574 (2)	0.227474 (17)	0.78759 (6)	0.03270 (12)	
N1	0.55104 (15)	0.27269 (10)	0.6504 (4)	0.0360 (8)	
N2	0.47521 (15)	0.28919 (10)	0.4458 (5)	0.0382 (7)	
C1	0.50711 (17)	0.26316 (13)	0.5388 (6)	0.0374 (9)	
H1	0.4979	0.2358	0.5246	0.045*	
C2	0.4883 (2)	0.32854 (13)	0.4677 (6)	0.0412 (9)	
H2	0.4672	0.3477	0.4048	0.049*	
C3	0.5320 (2)	0.34078 (14)	0.5813 (6)	0.0459 (11)	
H3	0.5410	0.3681	0.5959	0.055*	
C4	0.5623 (2)	0.31226 (13)	0.6730 (6)	0.0434 (10)	
H4	0.5912	0.3204	0.7526	0.052*	
N11	0.69371 (15)	0.24124 (11)	0.6493 (4)	0.0352 (8)	
C11	0.7500	0.2500	0.7228 (7)	0.0372 (12)	
H11	0.7500	0.2500	0.8339	0.045*	
C13	0.7500	0.2500	0.4059 (8)	0.061 (2)	
H13	0.7500	0.2500	0.2948	0.074*	
C14	0.6944 (2)	0.24140 (17)	0.4914 (6)	0.0499 (12)	
H14	0.6559	0.2355	0.4366	0.060*	
N21	0.57994 (17)	0.18287 (12)	0.6270 (5)	0.0434 (8)	
C21	0.56005 (17)	0.15562 (13)	0.5553 (5)	0.0381 (9)	
Se21	0.53008 (3)	0.114405 (17)	0.44108 (7)	0.06121 (16)	
N31	0.63231 (16)	0.26940 (12)	0.9575 (5)	0.0428 (8)	
C31	0.63448 (17)	0.29139 (13)	1.0643 (5)	0.0373 (9)	
Se31	0.63665 (3)	0.324181 (19)	1.23170 (7)	0.06461 (17)	
O41	0.66187 (15)	0.18255 (10)	0.9111 (4)	0.0522 (8)	
C41	0.6758 (4)	0.1790 (2)	1.0749 (8)	0.085 (2)	
H41A	0.6359	0.1747	1.1328	0.127*	
H41B	0.6964	0.2034	1.1118	0.127*	
H41C	0.7048	0.1566	1.0918	0.127*	
H1O4	0.6862	0.1792	0.8352	0.127*	

### Atomic displacement parameters (Å^2^)

**Table:**

	*U*^11^	*U*^22^	*U*^33^	*U*^12^	*U*^13^	*U*^23^
Co1	0.0252 (2)	0.0341 (3)	0.0388 (3)	−0.0009 (2)	−0.0010 (2)	−0.0043 (2)
N1	0.0287 (15)	0.0336 (18)	0.046 (2)	−0.0015 (13)	−0.0036 (13)	0.0000 (15)
N2	0.0294 (14)	0.0376 (17)	0.048 (2)	−0.0013 (14)	−0.0020 (14)	0.0017 (17)
C1	0.0270 (16)	0.036 (2)	0.049 (2)	0.0006 (15)	−0.0018 (18)	−0.0026 (19)
C2	0.045 (2)	0.033 (2)	0.045 (2)	0.0008 (17)	−0.0001 (19)	0.0031 (19)
C3	0.059 (3)	0.029 (2)	0.049 (3)	−0.0082 (19)	0.003 (2)	−0.0002 (19)
C4	0.048 (2)	0.039 (2)	0.043 (2)	−0.0022 (18)	−0.0087 (19)	−0.0029 (19)
N11	0.0268 (14)	0.0419 (19)	0.037 (2)	−0.0028 (13)	−0.0005 (13)	0.0017 (14)
C11	0.029 (2)	0.050 (3)	0.032 (3)	0.000 (2)	0.000	0.000
C13	0.046 (4)	0.108 (7)	0.030 (4)	−0.001 (4)	0.000	0.000
C14	0.0295 (19)	0.075 (3)	0.045 (3)	−0.005 (2)	−0.0032 (18)	−0.003 (2)
N21	0.0422 (18)	0.040 (2)	0.048 (2)	0.0000 (16)	−0.0047 (16)	−0.0091 (17)
C21	0.0304 (16)	0.043 (2)	0.042 (2)	0.0029 (16)	0.0016 (17)	0.002 (2)
Se21	0.0661 (3)	0.0505 (3)	0.0670 (4)	−0.0056 (2)	−0.0100 (3)	−0.0199 (3)
N31	0.0419 (18)	0.045 (2)	0.042 (2)	−0.0081 (16)	0.0024 (16)	−0.0071 (18)
C31	0.0276 (17)	0.039 (2)	0.046 (3)	−0.0020 (15)	0.0036 (16)	0.003 (2)
Se31	0.0715 (3)	0.0632 (4)	0.0592 (3)	−0.0041 (3)	0.0086 (3)	−0.0256 (3)
O41	0.0456 (16)	0.054 (2)	0.057 (2)	0.0146 (15)	−0.0044 (15)	0.0063 (16)
C41	0.101 (5)	0.071 (4)	0.082 (5)	0.002 (4)	−0.047 (4)	0.007 (3)

### Geometric parameters (Å, °)

**Table:**

Co1—N1	2.191 (3)	N11—C11	1.335 (4)
Co1—N2^i^	2.188 (3)	C11—N11^ii^	1.335 (4)
Co1—N11	2.184 (3)	C11—H11	0.9300
Co1—N21	2.064 (4)	C13—C14	1.372 (6)
Co1—N31	2.059 (4)	C13—C14^ii^	1.372 (6)
Co1—O41	2.142 (3)	C13—H13	0.9300
N1—C1	1.333 (5)	C14—H14	0.9300
N1—C4	1.342 (5)	N21—C21	1.157 (5)
N2—C1	1.331 (5)	C21—Se21	1.774 (5)
N2—C2	1.341 (5)	N31—C31	1.154 (5)
C1—H1	0.9300	C31—Se31	1.773 (4)
C2—C3	1.366 (6)	O41—C41	1.405 (7)
C2—H2	0.9300	O41—H1O4	0.8139
C3—C4	1.364 (7)	C41—H41A	0.9600
C3—H3	0.9300	C41—H41B	0.9600
C4—H4	0.9300	C41—H41C	0.9600
N11—C14	1.323 (6)		
			
N31—Co1—N21	176.69 (16)	C2—C3—H3	120.6
N31—Co1—O41	89.55 (15)	N1—C4—C3	121.2 (4)
N21—Co1—O41	87.46 (14)	N1—C4—H4	119.4
N31—Co1—N11	90.55 (13)	C3—C4—H4	119.4
N21—Co1—N11	90.75 (14)	C14—N11—C11	116.8 (4)
O41—Co1—N11	87.77 (13)	C14—N11—Co1	122.6 (3)
N31—Co1—N2^i^	87.12 (14)	C11—N11—Co1	120.5 (3)
N21—Co1—N2^i^	91.27 (14)	N11—C11—N11^ii^	125.1 (5)
O41—Co1—N2^i^	86.37 (13)	N11—C11—H11	117.5
N11—Co1—N2^i^	173.71 (13)	N11^ii^—C11—H11	117.5
N31—Co1—N1	92.13 (14)	C14—C13—C14^ii^	117.1 (6)
N21—Co1—N1	90.91 (14)	C14—C13—H13	121.5
O41—Co1—N1	177.16 (14)	C14^ii^—C13—H13	121.5
N11—Co1—N1	89.92 (12)	N11—C14—C13	122.1 (4)
N2^i^—Co1—N1	96.00 (13)	N11—C14—H14	118.9
C1—N1—C4	116.4 (4)	C13—C14—H14	118.9
C1—N1—Co1	123.3 (3)	C21—N21—Co1	169.9 (4)
C4—N1—Co1	120.3 (3)	N21—C21—Se21	178.7 (4)
C1—N2—C2	116.8 (4)	C31—N31—Co1	166.0 (3)
C1—N2—Co1^iii^	124.1 (3)	N31—C31—Se31	178.4 (4)
C2—N2—Co1^iii^	118.5 (3)	C41—O41—Co1	129.6 (4)
N2—C1—N1	125.9 (4)	C41—O41—H1O4	128.9
N2—C1—H1	117.0	Co1—O41—H1O4	92.4
N1—C1—H1	117.0	O41—C41—H41A	109.5
N2—C2—C3	120.8 (4)	O41—C41—H41B	109.5
N2—C2—H2	119.6	H41A—C41—H41B	109.5
C3—C2—H2	119.6	O41—C41—H41C	109.5
C4—C3—C2	118.9 (4)	H41A—C41—H41C	109.5
C4—C3—H3	120.6	H41B—C41—H41C	109.5

Symmetry codes: (i) −*x*+1, −*y*+1/2, *z*+1/2; (ii) −*x*+3/2, −*y*+1/2, *z*; (iii) −*x*+1, −*y*+1/2, *z*−1/2.
